# The impact of an intensive care telemedicine program on weaning from invasive ventilation: a secondary analysis of the ERIC trial

**DOI:** 10.1186/s13054-026-06234-z

**Published:** 2026-08-07

**Authors:** Moritz F. Adam, Julius J. Grunow, Andreas Edel, Karin Steinecke, Friederike S. Schuster, Claudia D. Spies, Björn Weiss, Nicolas Paul

**Affiliations:** Department of Anesthesiology and Intensive Care Medicine (CCM/CVK), Charité – Universitätsmedizin Berlin, corporate member of Freie Universität Berlin and Humboldt-Universität zu Berlin, Augustenburger Platz 1, Berlin, 13353 Germany

**Keywords:** ICU, Critical care, Mechanical ventilation, Telemedicine, Respiratory care, Weaning

## Abstract

**Background:**

ICU patients commonly require invasive mechanical ventilation. Although weaning from ventilation is crucial for patient outcomes, it is commonly not performed according to guideline recommendations. We investigated if a complex telemedicine intervention improves the weaning process and outcomes.

**Methods:**

This is a secondary analysis of the stepped-wedge cluster-randomized controlled Enhanced Recovery after Intensive Care (ERIC) trial, which was conducted among ten clusters of ICUs in the metropolitan area of Berlin, Germany. ERIC examined the impact of a complex telemedicine intervention, which included daily telemedicine rounding by an intensivist and ICU nurse, on the adherence to eight quality indicators of ICU care. We analyzed patients who received invasive mechanical ventilation for at least two consecutive days and at least one spontaneous breathing trial (SBT). We investigated the impact of the intervention on the weaning process and weaning outcomes using descriptive statistics and mixed-effects regressions.

**Results:**

Of 1,463 patients enrolled in the trial, 308 patients were analyzed (control condition: 55; intervention condition: 253). Patients in the intervention group received significantly more SBTs than patients in the control group (51% vs 27% of the patient days; *p* < 0.001). There was no difference between the groups with respect to the weaning classification (*p* = 0.21), weaning success rate (28% vs 36%; *p* = 0.32), and time from the first SBT to successful weaning (2 [IQR 1, 5] vs 4 [IQR 1, 13] days; *p* = 0.12). The median time of invasive mechanical ventilation of successfully weaned patients was significantly shorter in the intervention than in the control group (7 [IQR 4, 10] vs 9 [IQR 6, 20] days; *p* = 0.031). In our multivariable regressions, the intervention was not associated with weaning success (OR for weaning failure: 1.45 [95%-CI 0.71–2.95]; *p* = 0.302), but with a shorter time between the first SBT and successful weaning (β = 0.56 [0.32–0.98]; *p* = 0.042).

**Conclusions:**

Although ICU telemedicine did not improve the weaning success rate, our findings indicate that it facilitates the exploitation of existing weaning potential by ensuring more frequent SBTs and thereby shortening ventilation duration of successfully weaned patients. The study is the first to highlight the potential of telemedicine to enhance ICU weaning practices.

**Registration:**

www.ClinicalTrials.gov (Identifier: NCT03671447; first submitted on August 22, 2018).

**Graphical abstract:**

**Figure:**
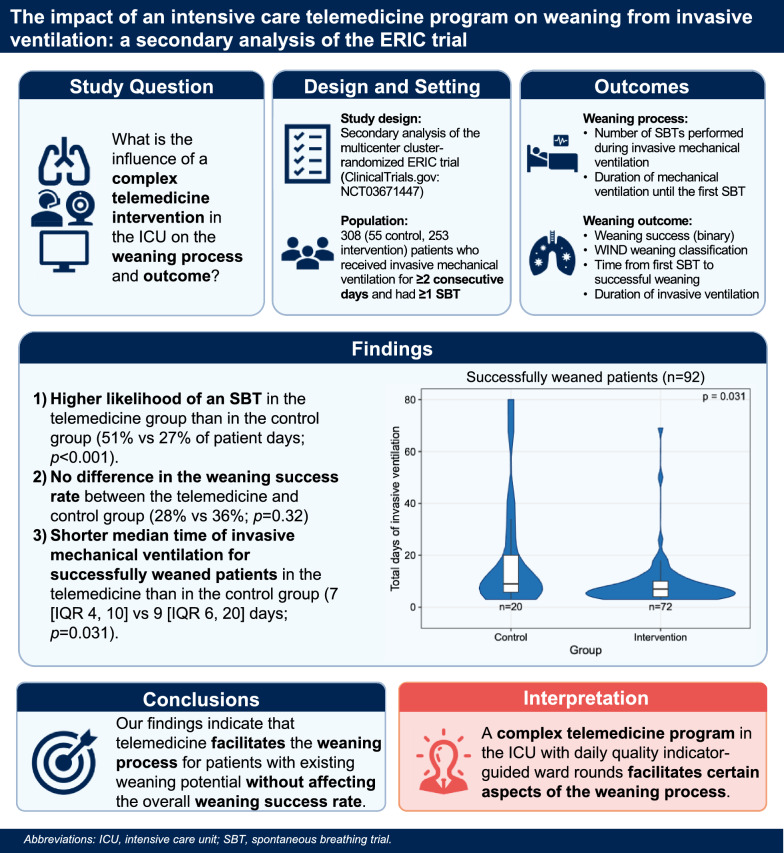

**Supplementary Information:**

The online version contains supplementary material available at 10.1186/s13054-026-06234-z.

## Background

Approximately one quarter of intensive care unit (ICU) patients requires invasive mechanical ventilation for a mean of almost four days [1]. Invasive mechanical ventilation is associated with detrimental outcomes such as higher mortality, longer hospital length of stay, a lower quality of life, and a higher risk for disability after hospital discharge [2–4]. Delays in initiating weaning from ventilation prolong its duration, increase weaning failure rates and decrease long-term survival [5, 6]. Weaning failure may lead to long-term invasive mechanical ventilation [7], which is associated with tremendous health care costs [8]. About one third of patients who were mechanically ventilated for at least two days was not weaned from ventilation at 90 days [6].

Weaning from invasive ventilation requires systematic assessments. There is evidence that protocol-based weaning can reduce the duration of mechanical ventilation [9]. Although knowledge gaps remain with respect to the most-effective weaning protocol [10], guidelines of the American Thoracic Society, the American College of Chest Physicians and the German Respiratory Society recommend that weaning protocols should include the following elements: a daily, objective assessment of readiness to wean, the early performance of an SBT, the assessment for extubation and the management of extubation failures [11–13]. SBTs should be performed under minimal sedation and with the help of ventilator modes that provide inspiratory pressure support [14–16]. As part of a set of quality indicators of acute ICU care, the German Interdisciplinary Association of Intensive Care and Emergency Medicine recommends the daily assessment for readiness to wean and daily SBTs using a standardized, guideline-based weaning protocol [17].

Despite its importance, the multinational WEAN SAFE study concluded that frequently, weaning is not conducted in accordance with guideline recommendations [6, 13, 18]. Telemedicine in critical care has emerged as a potential tool to facilitate the weaning process. It was shown that telemedicine support increases the bedside adherence to evidence-based practices, including the prevention of ventilator-associated pneumonia [19]. Moreover, telemedicine may improve weaning indirectly by having a bundle effect through improvements in early mobilization, ventilator settings as well as delirium and sedation management [12, 20, 21]. In the Enhanced Recovery after Intensive Care (ERIC) study, we demonstrated that a complex telemedicine intervention with daily, quality indicator-focused telemedicine rounding improves the adherence to process quality indicators, including global indicators on weaning, early mobilization, and the management of sedation, pain, and delirium [18, 19, 22].

The impact of a telemedicine intervention in the ICU on the process of weaning and weaning outcomes is unknown. Hence, in this secondary analysis of the ERIC trial, we investigated the influence of the complex telemedicine intervention on these outcomes.

## Methods

### Study design, setting and inclusion criteria

This study is a secondary analysis of the multicenter, stepped-wedge cluster-randomized controlled ERIC trial, which was conducted in Berlin and the surrounding state of Brandenburg in Germany (ClinicalTrials.gov: NCT03671447; first submitted on August 22, 2018) [22, 23]. Patients were enrolled in ten clusters, each consisting of up to three ICUs. Two clusters had ≤ 300 hospital beds, three clusters had 301 to 699 hospital beds, and five clusters had ≥ 700 hospital beds. Following the stepped-wedge cluster-randomized trial design, all clusters commenced enrolment under control conditions. In three pre-defined sequence groups, clusters transitioned to the intervention phase, in which enrolled patients received a complex telemedicine intervention. Patients could be enrolled if they had an expected ICU length of stay of ≥ 24 h, were ≥ 18 years old and had statutory health insurance coverage. The study received approval from the institutional review board of Charité - Universitätsmedizin Berlin (EA1/006/18, January 26, 2018) [22, 23].

In this secondary analysis, we assessed the influence of the study intervention on the process of weaning and weaning outcomes. Hence, as additional inclusion criteria for this analysis, we included all ERIC study patients who received invasive mechanical ventilation for at least two consecutive days and had at least one SBT.

### Telemedicine intervention and usual care

Patients that were enrolled under intervention conditions received daily telemedicine rounding by an experienced intensivist and ICU nurse of the telemedicine team located at Charité using a telemedicine cart (Figure S1). The telemedicine cart established a secure audio-visual connection between the telemedicine hub at Charité and the local ICU. The daily telemedicine rounding was guided by eight quality indicators of acute intensive care medicine by the German Interdisciplinary Association of Intensive Care and Emergency Medicine [18]. Beyond the quality indicators, additional recommendations could be given during rounding. The telemedicine team could provide advice, leaving the ultimate decisions and its legal implications with the local ICUs. In addition to scheduled rounds, the telemedicine team provided an around-the-clock and on-demand call service for participating clusters.

At the end of the control phase and prior to commencement of the telemedicine intervention, two to four staff members of each participating ICU cluster underwent a blended-learning program with nine e-learning modules, four simulation workshops and on-the-job training pertaining to the quality indicators and the telemedicine cart. Each e-learning module took 30–45 min and concluded with a multiple-choice exam. One e-learning module was on early weaning from invasive ventilation, where participants learnt how to conduct daily evaluations of invasive ventilation, readiness to wean and SBTs. In one simulation workshop given by respiratory therapists, physical therapists, nurses, and intensivists, participants underwent clinical case simulations on lung-protective ventilation, assessment of readiness to wean and conducting SBTs.

### Outcomes, definition of variables and data sources

In this secondary analysis, the outcomes of interest were, first, the weaning process and, second, the weaning outcomes. For the weaning process, we considered the following: the number of SBTs performed during invasive mechanical ventilation and the duration of mechanical ventilation until the first SBT. For the weaning outcomes, we considered the following: weaning success (binary), the weaning classification, the time from the first SBT to successful weaning, and the duration of invasive ventilation.

The primary analysis of the duration of invasive ventilation was restricted to successfully weaned patients, as the duration of invasive ventilation in patients with weaning failure is influenced by factors beyond the weaning process, such as death and timing of ICU discharge. Additionally, we analyzed the duration of invasive ventilation across all weaning classifications.

Invasive ventilation was defined as ventilation through an endotracheal tube or a tracheal cannula. All SBTs were protocol-based assessments, although the specific SBT protocols may have varied across study centers. Detailed information on the exact SBT modality was not collected. The weaning process was classified based on the Weaning according to a New Definition (WIND) classification [24] as either easy weaning (successful weaning within one day after the first SBT), difficult weaning (successful weaning within two to seven days after the first SBT), prolonged weaning (successful weaning after more than seven days after the first SBT), or weaning failure (no successful weaning). Following the WIND classification, death before successful liberation from mechanical ventilation was considered weaning failure. This binary definition of weaning success and weaning failure was chosen to ensure consistency with established weaning frameworks [24]. Following previous liberation studies considering a ventilator-free period of 48 to 72 h as successful weaning [9, 11, 25], weaning success was defined as a period of at least three consecutive days without ventilation through an endotracheal tube or a tracheal cannula and with no such ventilation on the last documented ICU day. We deviated from the WIND classification, which requires a period of at least seven consecutive days without ventilation for weaning success [24], because ventilation data was not available beyond ICU discharge, which frequently occurred within this period. To ensure that reintubation and weaning failure did not occur after the study period, patients that were in the study for fewer than three days following extubation were excluded. Weaning success was defined irrespective of tracheostomy placement during ICU treatment. If patients had more than one episode of ventilation with one or more SBT followed by a successful weaning, we only considered the first episode. If patients were admitted to the ICU and were enrolled in the ERIC trial more than once, we analyzed their first ICU treatment.

At ICU admission, we collected demographic variables, admission diagnosis, admission source, the Simplified Acute Physiology Score (SAPS) II, and the Sequential Organ Failure Assessment (SOFA) score. Over the course of the ICU stay, we documented the current airway for each day and if an SBT was conducted.

### Statistics

Continuous variables are presented as mean (standard deviation) or median (interquartile range [IQR]), depending on the distribution. Categorical variables are presented as absolute (n) and relative (%) frequencies. We used Pearson’s Chi-squared tests, Fisher’s exact tests or Fisher’s exact test with Monte Carlo simulation to compare categorical variables between patients enrolled under control and intervention conditions. Depending on the distribution, either Mann-Whitney U tests or t-tests were used to compare continuous variables between groups. Normal distribution was visually checked using histograms. We analyzed the time to successful weaning after the first SBT using Kaplan-Meier estimators and log-rank tests for patients who achieved successful weaning. To assess determinants of weaning failure, we employed a multivariable mixed-effects logistic regression model with weaning failure as dependent variable, the study group, age, delirium during invasive ventilation, SAPS II, SOFA, binary variables for respiratory and neurologic admission diagnoses as fixed effects, and the study cluster as random effect. In a second regression model, we analyzed the determinants of the time between the first SBT and successful weaning (dependent variable) for successfully weaned patients. For this purpose, we employed a gamma generalized linear mixed-effects model with a log link, with the study group, age, delirium during invasive ventilation, SAPS II and SOFA as fixed effects, and the study cluster as random effect. We considered *p* ≤ 0.05 to be statistically significant. Analyses were performed using R (version 4.5.0, 2025-04-11 ucrt) in RStudio (Posit Software, PBC, Boston, MA) with the packages lme4 (version 1.1.38) and lmerTest (version 3.1.3).

## Results

### Study population

**Fig. 1:**
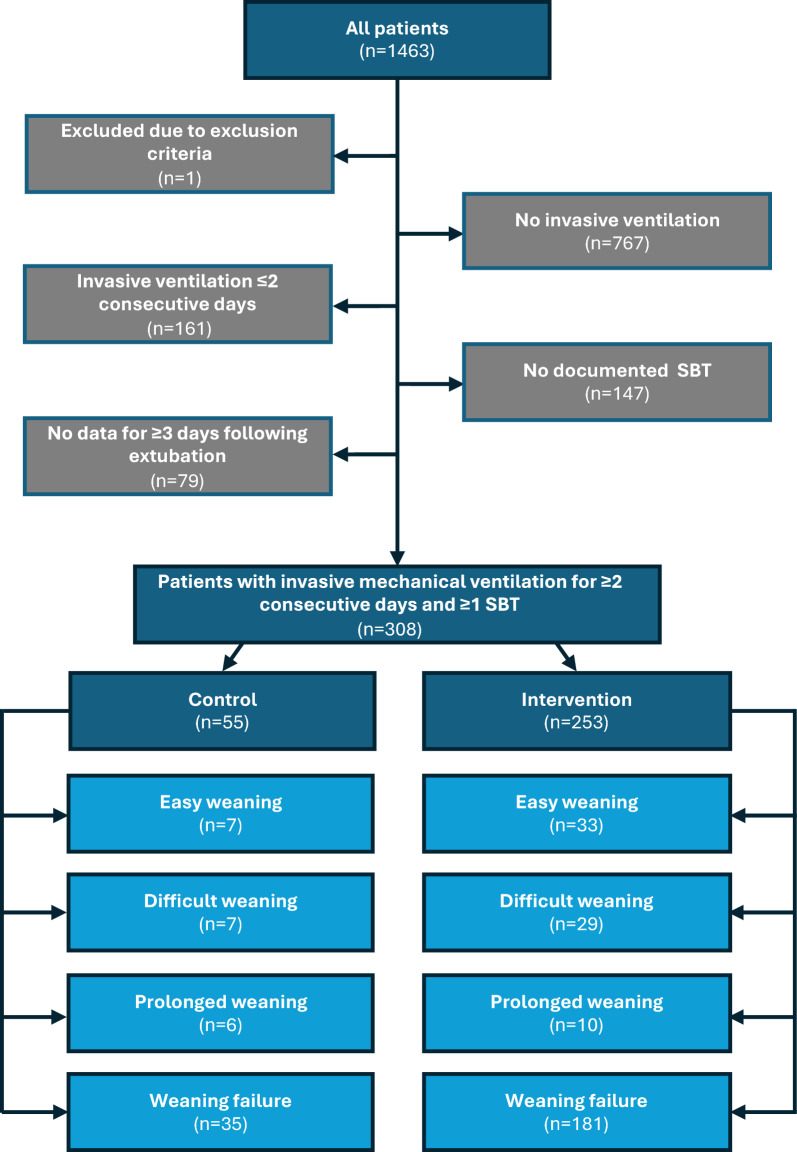
Study inclusion flowchart. Easy weaning: ≤ 1 day between first SBT and successful weaning. Difficult weaning: 2 to 7 days between the first SBT and successful weaning. Prolonged weaning: ≥ 8 days between first SBT and successful weaning. Weaning failure: death before successful weaning or discharged with invasive airway. SBT, spontaneous breathing trial

In the ERIC trial, ten participating clusters enrolled 1,463 patients between September 4, 2018, and March 31, 2020. Of those, 308 patients received invasive mechanical ventilation for at least two consecutive days and underwent at least one SBT, with 55 of these patients treated under control conditions and 253 patients treated under intervention conditions (telemedicine) (Fig. 1). About half of all patients were recruited in two academic clusters (Table S1). The patient characteristics of both groups were similar except for a higher age in the control than in the intervention group (72 [IQR 63.5, 80] vs 69 [IQR 56, 78] years; *p* = 0.024) (Table 1).

**Table 1:** Baseline characteristics of the study population

Variable	Control	Intervention	All patients	*p*
	**(n = 55)**	**(n = 253)**	**(n = 308)**	
**Age**	72 [63.5, 80]	69 [56, 78]	70 [58, 78]	**0.024**
**Female**	22 (40%)	94 (37%)	116 (38%)	0.809
**Body mass index, kg/m**^**2**^	27.3 [24.4, 31.8]	26.1 [23.1, 31.1] (n = 252)	26.2 [23.4, 31.1] (n = 307)	0.372
**Admission source**				
Other ICU	5 (9%)	25 (10%)	30 (10%)	0.328
External	3 (6%)	32 (13%)	35 (11%)	
Normal ward	11 (20%)	29 (13%)	40 (13%)	
Surgical	20 (36%)	93 (37%)	113 (37%)	
Emergency medical service	16 (29%)	74 (29%)	90 (29%)	
**Admission reason**		(n = 250)	(n = 305)	
Elective surgery	8 (15%)	40 (16%)	48 (16%)	0.966^1^
Medical	27 (49%)	116 (46%)	143 (47%)	
Emergency surgery	20 (36%)	94 (38%)	114 (37%)	
**Primary ICU admission diagnosis**				
Cardiovascular disorder	10 (18%)	63 (25%)	73 (24%)	0.276^2^
Gastrointestinal disorder	6 (11%)	19 (8%)	25 (8%)	
Metabolic or endocrine disorder	2 (4%)	5 (2%)	7 (2%)	
Neurologic disorder	7 (13%)	40 (16%)	47 (15%)	
Oncologic disorder	4 (7%)	23 (9%)	27 (9%)	
Other	1 (2%)	2 (1%)	3 (1%)	
Respiratory disorder	11 (20%)	25 (10%)	36 (12%)	
Sepsis/Infection	12 (22%)	51 (20%)	63 (20%)	
Trauma	2 (4%)	25 (10%)	27 (9%)	
**SAPS II at admission**	46.7 (17) (n = 54)	47 (18.2) (n = 251)	46.9 (18) (n = 305)	0.926^3^
**SOFA score at admission**	8 [3.5, 10]	9 [6, 11]	8 [6, 11]	0.061
**ICU mortality**	14 (25%) (n = 55)	51 (20%) (n = 249)	65 (21%) (n = 304)	0.490

Median [Q1, Q3], mean (SD) or n (%), n below the respective total group size is indicated in parentheses. Groups were compared using Pearson’s Chi-squared tests or Mann-Whitney U tests unless specified otherwise. *p* ≤ 0.05 is highlighted in bold. ^1^Fisher’s exact test, ^2^Fisher’s exact test with Monte Carlo simulation, ^3^t-test. BMI, body mass index; ICU, intensive care unit; SAPS II, Simplified Acute Physiology Score II; SOFA, Sequential Organ Failure Assessment

Compared with patients included in our analysis, patients who received ≥ 2 consecutive days of invasive ventilation but were excluded due to the absence of an SBT had a higher ICU mortality, a lower SOFA score at admission (Table S2), and were more frequently in the control group (Table S3).

### The weaning process

The 55 patients treated under control conditions had a total of 1,065 patient days with invasive mechanical ventilation. On 283 (27%) of those, an SBT was performed. The 253 patients treated under intervention conditions had a total of 4,890 patient days with invasive mechanical ventilation. The proportion of days with an SBT (n = 2473/4890 [51%] days; *p* < 0.001) was significantly higher in the intervention group (Table S4). Additionally, the number of SBTs per patient was higher in the intervention than in the control group (5 [IQR 2, 14] vs 2 [IQR 1, 4.5]; *p* = 0.001) (Table S5). The median time to the first SBT following initiation of invasive mechanical ventilation was 3 [IQR 1, 6.5] days in the control group and 2 [IQR 1, 5] days in the intervention group (*p* = 0.393) (Table S6).

### The weaning outcomes

Of the 55 patients treated under control conditions, 35 patients (64%) had weaning failure and 20 patients (36%) were successfully weaned; 7 patients (13%) were classified as easy weaning, 7 patients (13%) as difficult weaning, and 6 patients (11%) as prolonged weaning (Table 2). Of the 253 patients treated under intervention conditions, 181 patients (72%) had weaning failure and 72 patients (28%) were successfully weaned; 33 patients (13%) patients were classified as easy weaning, 29 patients (11%) as difficult weaning, and 10 patients (4%) as prolonged weaning. The differences in weaning classification between the study groups were not significant (*p* = 0.21). There was also no significant difference in the weaning success rate (*p* = 0.32). Compared to patients with successful weaning, weaning failure patients had neurologic admission diagnoses more frequently (*p* = 0.003) (Table S7). We did not find differences in weaning classification between academic and non-academic study clusters (*p* = 0.169) (Table S8).

**Table 2:** Weaning classification according to the weaning according to a new definition (WIND) classification [24]

WIND classification	Control	Intervention	All patients	*p*
	**(n = 55)**	**(n = 253)**	**(n = 308)**	
Easy	7 (13%)	33 (13%)	40 (13%)	0.21
Difficult	7 (13%)	29 (11%)	36 (12%)	
Prolonged	6 (11%)	10 (4%)	16 (5%)	
Failure	35 (64%)	181 (72%)	216 (70%)	

n (%). Groups were compared using Fisher’s exact test

**Fig. 2:**
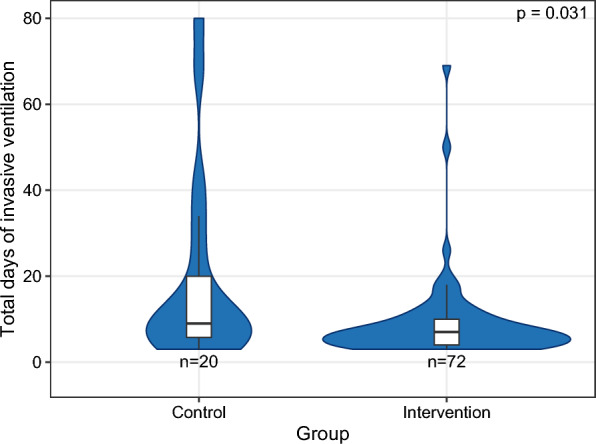
Days of invasive ventilation for successfully weaned patients (n = 92). In the control group, 20/55 patients were successfully weaned. In the intervention group, 72/253 patients were successfully weaned. In total, 92/308 patients were successfully weaned. Groups were compared using Mann-Whitney U test. SBT, spontaneous breathing trial

Among successfully weaned patients (control group: 20/55 patients; intervention group: 72/253 patients), there was no significant difference in the time from the first SBT to successful weaning between the intervention and control groups (2 [IQR 1, 5] vs 4 [IQR 1, 13] days, *p* = 0.12). There was also no significant difference in the Kaplan-Meier curves of the cumulative proportion of successfully weaned patients for both groups (*p* = 0.13) (Figure S2). Among successfully weaned patients, the duration of invasive ventilation was significantly shorter in the intervention group compared to the control group (7 [IQR 4, 10] vs 9 [IQR 6, 20] days; *p* = 0.031) (Fig. 2). There was no difference in the duration of invasive ventilation between study groups when comparing each weaning classification or all patients (including weaning failure) (Table S9).

### Determinants of weaning failure and the time from the first SBT to successful weaning

The multivariable mixed-effects logistic regression model predicting weaning failure among all patients showed no significant influence of the study group on weaning failure (OR intervention group: 1.45 [95%-CI 0.71–2.95]; *p* = 0.302). A neurologic admission diagnosis was associated with a significantly higher risk for weaning failure (OR 7.74 [95%-CI 2.27–26.40]; *p* = 0.001) (Table 3). The gamma generalized linear mixed-effects regression model with a log link predicting the time between first SBT and successful weaning among those successfully weaned showed that the intervention was associated with a significantly shorter time between the first SBT and successful weaning (β = 0.56 [95%-CI 0.32–0.98]; *p* = 0.042). Delirium during mechanical ventilation was associated with a significantly longer time between the first SBT and successful weaning (β = 2.23 [95%-CI 1.37–3.63]; *p* = 0.002) (Table 4).

**Table 3:** Multivariable mixed-effects logistic regression model predicting weaning failure among all patients

Predictors	Odds ratio	95%-CI	*p*
Study group, intervention	1.45	0.71 – 2.95	0.302
Age, years	1.01	0.99 – 1.03	0.449
Delirium during ventilation, yes	1.25	0.70 – 2.22	0.454
SAPS II	1.01	0.99 – 1.02	0.486
SOFA score	0.98	0.91 – 1.05	0.522
Respiratory disorder, yes	1.39	0.61 – 3.19	0.433
Neurologic disorder, yes	7.74	2.27 – 26.40	**0.001**
Intercept	0.61	0.14 – 2.74	0.518
Random effects			
σ^2^	3.29		
τ_00 study cluster_	0.17		
ICC	0.05		
N _study cluster_	10		
Observations	305		
Marginal R^2^ / conditional R^2^	0.141 / 0.182		

*p* ≤ 0.05 is highlighted in bold. ICC, intraclass correlation coefficient; SAPS II, Simplified Acute Physiology Score II; SOFA, Sequential Organ Failure Assessment

**Table 4:** Gamma generalized linear mixed-effects regression model with a log link predicting the time between first spontaneous breathing trial and successful weaning from invasive mechanical ventilation (in days) among successfully weaned patients

Predictors	Coefficient (β)	95%-CI	*p*
Study group, intervention	0.56	0.32 – 0.98	**0.042**
Age, years	0.99	0.98 – 1.01	0.320
Delirium during ventilation, yes	2.23	1.37 – 3.63	**0.002**
SAPS II	0.99	0.98 – 1.01	0.287
SOFA score	1.02	0.97 – 1.08	0.468
Intercept	10.45	2.91 – 37.53	** < 0.001**
Random Effects			
σ^2^	0.79		
τ_00 study cluster_	0.48		
ICC	0.38		
N _study cluster_	10		
Observations	92		
Marginal R^2^ / conditional R^2^	0.145 / 0.466		

*p* ≤ 0.05 is highlighted in bold. ICC, intraclass correlation coefficient; SAPS II, Simplified Acute Physiology Score II; SOFA, Sequential Organ Failure Assessment

## Discussion

This secondary analysis of a large ICU trial showed that a complex telemedicine intervention focusing on procedural quality did not affect the overall weaning success rate or weaning classification but might have a positive impact on some aspects of the weaning process. The intervention was associated with a higher share of patient days with an SBT and a shorter duration of invasive ventilation among successfully weaned patients. Further, we found a shorter time from the first SBT to successful weaning in our multivariable regression, but not in the descriptive analysis.

Our analysis is the first in-depth investigation on how a complex telemedicine intervention may affect the weaning process in the ICU. It indicates that regular structured telemedicine ward rounds can improve the standardized weaning process via constant monitoring and evaluation, allowing SBTs to be performed more frequently. This may have shortened the duration of mechanical ventilation for those who have been weaned successfully. Although the details of weaning protocols vary between studies, readiness to wean assessments and SBTs are key elements of many protocols shown to improve ventilation outcomes [9] and recommended core elements of German and American guidelines [12, 13]. However, as the overall weaning success rate was unaffected, our findings suggest that the intervention facilitated earlier and more rigorous weaning only for patients with existing weaning potential. The lack of an effect on the weaning success rate may also be explained by the fact that half of our patients were recruited at academic centers where the standard of care may have been high already [10, 11].

Although the influence of ICU telemedicine on the weaning process and outcomes has not been explored, two cluster-randomized controlled trials investigated the effect of ICU telemedicine on ventilation outcomes [22, 26]. In the Brazilian TELESCOPE trial, participating ICUs received daily multidisciplinary telemedicine ward rounds, training on evidence-based treatment protocols (including mechanical ventilation), and monthly feedback on performance indicators [26]. The study did not show any significant improvement of ventilator-associated events and ventilator-free days at 28 days [26]. However, compared to our analysis, the TELESCOPE cohort was younger and had a lower SOFA score at admission. This may indicate that a beneficial effect of a telemedicine ward rounds on the weaning process may be limited to patients with a higher severity of illness. The second trial is the ERIC study, which we performed this secondary analysis from [22]. The primary analysis of the ERIC trial focused on the adherence to eight indicators of procedural quality [18, 22], one of which pertained to the global weaning process (i.e., indication for mechanical ventilation, readiness to wean assessments, and SBT performance). The analysis found a positive intervention effect on the quality indicator adherence [22], so our in-depth analysis of weaning complements findings of the primary analysis. Other studies on ICU telemedicine employed before-after designs and varied with respect to the telemedicine intervention [27]. In a single-center before-after study from the US, the telemedicine program was associated with increased adherence to best practices to prevent ventilator-associated pneumonia and a shorter duration of mechanical ventilation [21]. These findings are in line with our study, showing that ICU telemedicine may be effective in improving evidence-based practices for mechanical ventilation.

The ERIC intervention combined daily telemedicine ward rounds with a blended-learning program for local ICU staff, including training on early weaning from invasive ventilation. Our observed effects on the weaning process can therefore not be attributed to telemedicine alone but may also reflect effects of the preceding training. This is supported by evidence that training of local champions and continuous feedback increases the adherence to daily spontaneous awakening and breathing trials, accompanied by decreases in duration of mechanical ventilation [28]. In two other single-center before-after studies, the education of ICU staff on a structured weaning protocol was associated with increased protocol adherence [29, 30]. However, education is not invariably sufficient, as shown in another study where protocol adherence and ventilation duration remained unchanged despite extensive training [31]. Ultimately, our analysis cannot disentangle the independent contributions of the training and telemedicine. Existing literature has similar limitations, as the TELESCOPE trial also implemented training alongside telemedicine [26]. Potentially, structured telemedicine rounding may help maintain the increased adherence to weaning protocols induced by preceding training, as it was shown that the positive effect of training alone subsides over time [29].

Findings from ERIC also showed improvements in early mobilization and the management of sedation, pain and delirium [22], underscoring the ability of telemedicine as a holistic quality improvement tool. In line with previous weaning studies [32, 33], delirium was associated with a prolonged time to successful weaning in our cohort, suggesting that improvements in delirium management could represent another potential pathway through which telemedicine facilitates weaning. The observed association between a neurological ICU admission diagnosis and weaning failure is also consistent with previous literature [6, 34, 35] and likely reflects the challenges of liberation from ventilation in patients with neurological impairments.

Strengths of this analysis include, first, the use of data from a multicenter cluster-randomized clinical trial that examined a broad spectrum of medical, surgical and mixed ICU patients. Second, the analysis of weaning was based on the internationally used WIND classification [24]. Study limitations include, first, the small sample size, as we analyzed a subgroup of the trial’s study population. Second, our analysis included more patients in the intervention than in the control group, raising concerns of residual confounding. This imbalance resulted from the stepped-wedge design of the parent trial, in which more patients were enrolled in the later trial phases when all clusters recruited under intervention conditions. Third, as the ICU data was collected once per day, we were unable to assess the hours between intubation, first SBT and successful weaning. Fourth, we did not collect any data about patients’ readiness to wean, which impedes the assessment of whether performing an SBT would have been appropriate on a particular day. To mitigate this limitation, we included only mechanically ventilated patients with at least one SBT. However, compared to our study cohort, the share of control group patients was higher among ventilated patients excluded due to the absence of an SBT. This suggests that the intervention itself may have increased the likelihood of performing an SBT, indicating a potential selection bias. Nevertheless, the time from initiation of mechanical ventilation to the first SBT was similar between groups, arguing against a major difference in the timing of weaning initiation. Fifth, our study is a secondary analysis of a large ICU trial which was not powered to analyze improvements in the weaning process. Hence, our findings should be regarded as hypotheses-generating for future prospective trials focusing on the impact of telemedicine on weaning.

## Conclusions

Our analysis suggests that a complex, structured telemedicine program in the ICU with daily quality indicator-guided ward rounds did not improve the weaning success rate but may facilitate certain aspects of the weaning process. The telemedicine intervention was associated with more SBTs and a reduced length of mechanical ventilation among successfully weaned patients. Hence, our findings indicate that telemedicine expedites the weaning process for patients with existing weaning potential without affecting the overall weaning success rate. We acknowledge, however, that further prospective analyses are needed to assess the effect of ICU telemedicine on weaning outcomes.

## Supplementary Information

Additional file 1.

Additional file 2.

## Data Availability

The datasets used and/or analyzed during the current study are available from the corresponding author upon reasonable scientific and non-commercial request.
