# Supplementary material for: The impact of an intensive care telemedicine program on weaning from invasive ventilation: a secondary analysis of the ERIC trial
Source: Crit Care. 2026 Aug 7;30:405. doi: 10.1186/s13054-026-06234-z (PMC13449835; doi:10.1186/s13054-026-06234-z)
Supplement: Supplementary file 1 [file 13054_2026_6234_MOESM1_ESM.docx]

**Additional file 1**

**Figure S1.** Telemedicine cart employed for establishing the audio-visual connection for telemedicine ward rounds during the intervention (Lite V3 from InTouch Health, now Teladoc Health).

From Figure S1 of Spies CD, et al. Effectiveness of an intensive care telehealth programme to improve process quality (ERIC): a multicentre stepped wedge cluster randomised controlled trial. Intensive Care Med. 2023;49(2):191–204.

**Figure S2.**  Kaplan-Meier estimators showing the cumulative proportion of successfully weaned patients by the days from the first SBT, for successfully weaned patients in the control group (n=20) and the intervention group (n=72).

In the control group, 20/55 patients were successfully weaned. In the intervention group, 72/253 patients were successfully weaned. Groups were compared using the log-rank test. SBT, spontaneous breathing trial.

**Table S1.** Total number of patients per ICU cluster participating in the ERIC trial (n=308).

| **Study cluster** | **Control** | **Intervention** | **All patients** |
| --- | --- | --- | --- |
|  | **(n=55)** | **(n=253)** | **(n=308)** |
| 1 | 3 (5%) | 13 (5%) | 16 (5%) |
| 2 | 5 (9%) | 1 (0%) | 6 (2%) |
| 3 | 10 (18%) | 14 (6%) | 24 (8%) |
| 4 | 3 (5%) | 26 (10%) | 29 (9%) |
| 5 | 1 (2%) | 2 (1%) | 3 (1%) |
| 6 | 8 (15%) | 66 (26%) | 74 (24%) |
| 7 | 11 (20%) | 66 (26%) | 77 (25%) |
| 8 | 6 (11%) | 14 (6%) | 20 (6%) |
| 9 | 0 (0%) | 20 (8%) | 20 (6%) |
| 10 | 8 (15%) | 31 (12%) | 39 (13%) |

n (%). In total, 12 clusters were randomized, but one cluster dropped out prior to trial commencement and another cluster dropped out without recruiting any patients. ICU, intensive care unit.

**Table S2.** Baseline characteristics of patients included in our analysis (n=308) compared to patients who received ≥2 consecutive days of invasive ventilation but were excluded due to the absence of an SBT (n=147).

| **Variable** | **Included in our study** | **Excluded due to absence of SBT** | **All patients** | ***p*** |
| --- | --- | --- | --- | --- |
|  | **(n=308)** | **(n=147)** | **(n=455)** |  |
| **Age** | 70 [58, 78] | 69 [58, 79] | 70 [58, 79] | 0.850 |
| **Female** | 116 (38%) | 59 (40%) | 175 (39%) | 0.686 |
| **Body mass index, kg/m^2^** | 26.2 [23.4, 31.1] (n=307) | 26.1 [23, 29.4] | 26.2 [23.4, 31.1] (n=454) | 0.227 |
| **Admission source** |  |  |  |  |
| Other ICU | 30 (10%) | 14 (10%) | 44 (10%) | 0.677 |
| External | 35 (11%) | 16 (11%) | 51 (11%) |  |
| Normal ward | 40 (13%) | 20 (14%) | 60 (13%) |  |
| Surgical | 113 (37%) | 45 (31%) | 158 (35%) |  |
| Emergency medical service | 90 (29%) | 52 (35%) | 142 (31%) |  |
| **Admission reason** | (n=305) | (n=144) | (n=449) |  |
| Elective surgery | 48 (16%) | 17 (12%) | 48 (14%) | 0.393^1^ |
| Medical | 143 (46%) | 77 (52%) | 143 (48%) |  |
| Emergency surgery | 114 (37%) | 50 (34%) | 114 (36%) |  |
| **ICU primary admission diagnosis** |  |  |  |  |
| Cardiovascular disorder | 73 (24%) | 30 (20%) | 103 (23%) | 0.968^2^ |
| Gastrointestinal disorder | 25 (8%) | 13 (9%) | 38 (8%) |  |
| Metabolic or endocrine disorder | 7 (2%) | 1 (1%) | 8 (2%) |  |
| Neurologic disorder | 47 (15%) | 24 (16%) | 71 (16%) |  |
| Oncologic disorder | 27 (9%) | 12 (8%) | 39 (9%) |  |
| Other | 3 (1%) | 1 (1%) | 4 (1%) |  |
| Respiratory disorder | 36 (12%) | 18 (12%) | 54 (12%) |  |
| Sepsis/Infection | 63 (21%) | 33 (22%) | 96 (21%) |  |
| Trauma | 27 (9%) | 15 (10%) | 42 (9%) |  |
| **SAPS II at admission** | 46.9 (18) (n=305) | 44.5 (17.1) (n=145) | 46.1 (17.7) (n=450) | 0.173^3^ |
| **SOFA score at admission** | 8 [6, 11] | 7 [5, 10] | 8 [5.5, 11] | **0.014** |
| **ICU mortality** | 65 (21%) (n=304) | 49 (33%) (n=146) | 114 (25%) (n=450) | **0.007** |

Median [Q1, Q3], mean (SD) or n (%), n below the respective total group size is indicated in parentheses. Groups were compared using Pearson’s Chi-squared tests or Mann-Whitney U tests unless specified otherwise. ^1^Fisher’s exact test, ^2^Fisher’s exact test with Monte Carlo simulation, ^3^t-test. BMI, body mass index; ICU, intensive care unit; SAPS II, Simplified Acute Physiology Score II; SOFA, Sepsis-related organ failure assessment.

**Table S3.** Study group distribution among patients included in our study (n=308) and patients who received ≥2 consecutive days of invasive ventilation but were excluded due to the absence of an SBT (n=147).

| **Study group** | **Included in our study**  **(n=308)** | **Excluded due to absence of SBT**  **(n=147)** | **All patients**  **(n=455)** | ***p*** |
| --- | --- | --- | --- | --- |
| Control | 55 (18%) | 61 (41%) | 116 (25%) | **<0.001** |
| Intervention | 253 (82%) | 86 (59%) | 339 (75%) |  |

n (%). Groups were compared using Pearson’s Chi-squared test. SBT, spontaneous breathing trial.

**Table S4.** Total number of patient days with invasive ventilation with a spontaneous breathing trial (n=308 patients with 5,955 patient days on invasive mechanical ventilation).

| **SBT performed** | **Control**  **(n=55 patients with 1,065 patient days)** | **Intervention**  **(n=253 patients with 4,890 patient days)** | **All patients**  **(n=308 patients with 5,955 patient days)** | ***p*** |
| --- | --- | --- | --- | --- |
| Yes | 283 (27%) | 2473 (51%) | 2756 (46%) | **<0.001** |
| No | 782 (73%) | 2417 (49%) | 3199 (54%) |  |

n (%). Groups were compared using Pearson’s Chi-squared test. SBT, spontaneous breathing trial. **Table S5.** Spontaneous breathing trials per patient (n=308).

| **Variable** | **Control** | **Intervention** | **All patients** | ***p*** |
| --- | --- | --- | --- | --- |
|  | **(n=55)** | **(n=253)** | **(n=308)** |  |
| Count of SBTs per patient | 2 [1, 4.5] | 5 [2, 14] | 4 [1, 12] | **0.001** |

Median [Q1, Q3]. Groups were compared using Mann-Whitney U test. SBT, spontaneous breathing trial.

**Table S6.** Number of patient days with invasive ventilation before the first spontaneous breathing trial (n=308).

| **Variable** | **Control** | **Intervention** | **All patients** | ***p*** |
| --- | --- | --- | --- | --- |
|  | **(n=55)** | **(n=253)** | **(n=308)** |  |
| Time of ventilation before first SBT | 3 [1, 6.5] | 2 [1, 5] | 2 [1, 5] | 0.393 |

Median [Q1, Q3]. Groups were compared using Mann-Whitney U test. SBT, spontaneous breathing trial.

**Table S7.** Distribution of admission diagnoses for patients with and without weaning failure (n=308).

| **Primary ICU admission**  **diagnosis** | **Successful weaning**  **(n=92)** | **Weaning failure**  **(n=216)** | **All patients (n=308)** | ***p*** |
| --- | --- | --- | --- | --- |
| Cardiovascular disorder | 28 (30%) | 45 (21%) | 73 (24%) | **0.003** |
| Gastrointestinal disorder | 10 (11%) | 15 (7%) | 25 (8%) |  |
| Metabolic or endocrine disorder | 3 (3%) | 4 (2%) | 7 (2%) |  |
| Neurologic disorder | 3 (3%) | 44 (20%) | 47 (15%) |  |
| Oncologic disorder | 10 (11%) | 17 (8%) | 27 (9%) |  |
| Respiratory disorder | 10 (11%) | 26 (12%) | 36 (12%) |  |
| Sepsis/Infection | 22 (24%) | 41 (19%) | 63 (21%) |  |
| Trauma | 5 (5%) | 22 (10%) | 27 (9%) |  |
| Other | 1 (1%) | 2 (1%) | 3 (1%) |  |

n (%). Groups were compared using Fisher’s exact test. ICU, intensive care unit.

**Table S8.** Weaning classification according to the Weaning according to a New Definition (WIND) classification, stratified by academic (n=151) and non-academic medical centers (n=157).

| **WIND classification** | **Non-academic study center**  **(n=157)** | **Academic study center**  **(n=151)** | **All patients (n=308)** | ***p*** |
| --- | --- | --- | --- | --- |
| Easy | 25 (16%) | 15 (10%) | 40 (13%) | 0.169 |
| Difficult | 18 (12%) | 18 (12%) | 36 (12%) |  |
| Prolonged | 11 (7%) | 5 (3%) | 16 (5%) |  |
| Failure | 103 (66%) | 113 (75%) | 216 (70%) |  |

n (%). Groups were compared using Fisher’s exact test. The weaning classification follows Beduneau G et al. Epidemiology of Weaning Outcome according to a New Definition. The WIND Study. Am J Respir Crit Care Med. 2017;195(6):772–83.

**Table S9.** Days of invasive ventilation for all patients and stratified by the Weaning according to a New Definition (WIND) classification (n=308).

| **WIND classification** | **Control**  **(n=55)** | **Intervention**  **(n=253)** | **All patients**  **(n=308)** | ***p*** |
| --- | --- | --- | --- | --- |
| All patients | 12 [6, 24] | 12 [6, 28] | 12 [6, 28] | 0.994 |
| Easy | 4 [4, 8.5] | 6 [3, 7] | 6 [3, 7.2] | 0.443 |
| Difficult | 9 [6, 10] | 6 [5, 9] | 6.5 [5, 9.2] | 0.197 |
| Prolonged | 38 [28, 61.5] | 17.5 [14, 24.5] | 23 [16.2, 44] | 0.050 |
| Failure | 16 [7, 24.5] | 20 [7, 32] | 20 [7, 31] | 0.208 |

Median [Q1, Q3]. Groups were compared using Mann-Whitney U test. The weaning classification follows Beduneau G et al. Epidemiology of Weaning Outcome according to a New Definition. The WIND Study. Am J Respir Crit Care Med. 2017;195(6):772–83.
